# Probiotic Properties of *Lactiplantibacillus plantarum* LB5 Isolated from Kimchi Based on Nitrate Reducing Capability

**DOI:** 10.3390/foods9121777

**Published:** 2020-11-30

**Authors:** Hyejin Sohn, You Hyun Chang, Jong Hyeok Yune, Chang Hee Jeong, Dong Min Shin, Hyuk Cheol Kwon, Do Hyun Kim, Sung Wook Hong, Hyelyeon Hwang, Jong Youn Jeong, Sung Gu Han

**Affiliations:** 1 Department of Food Science and Biotechnology of Animal Resources, Konkuk University, Seoul 05029, Korea; sonhjin123@konkuk.ac.kr (H.S.); hyunee601@konkuk.ac.kr (Y.H.C.); skyun0423@konkuk.ac.kr (J.H.Y.); hello01@konkuk.ac.kr (C.H.J.); s900704@konkuk.ac.kr (D.M.S.); rnjs1024@konkuk.ac.kr (H.C.K.); secret311@konkuk.ac.kr (D.H.K.); 2 World Institute of Kimchi, Gwangju 61755, Korea; swhong@wikim.re.kr (S.W.H.); hyelyeon@wikim.re.kr (H.H.); 3 School of Food Biotechnology and Nutrition, Kyungsung University, Busan 48434, Korea; jeongjy@ks.ac.kr

**Keywords:** *Lactiplantibacillus plantarum* LB5, nitrate reduction, kimchi, probiotic properties, safety assessment, antioxidant activity, anti-inflammatory activity

## Abstract

The purpose of this study was to investigate the probiotic properties of lactic acid bacteria isolated from Korean radish water kimchi (dongchimi). A total of 800 isolates of lactic acid bacteria were isolated from kimchi, and the strain having reduction and tolerance capability for nitrate and nitrite was selected and identified as *Lactiplantibacillus plantarum* LB5 (LPLB5) by 16S rRNA sequencing. LPLB5 showed higher tolerance to acidic pH values (pH 2.5), 0.3% bile salts, and heat treatment (40, 50, and 60 °C). Antibacterial activity showed strong inhibition against four food-borne pathogenic bacteria (*E. coli* O157:H7 ATCC 35150, *Pseudomonas aeruginosa* KCCM 12539, *Listeria monocytogenes* KCCM 40307, and *Staphylococcus aureus* ATCC 25923). The strain did not show any antibiotic resistance, β-hemolytic activity, or ability to produce β-glucuronidase. LPLB5 also exhibited a 30% auto-aggregation ability and 33–60% co-aggregation ability with four pathogenic bacteria (*E. coli* O157: H7 ATCC 35150, *E. coli* KCTC 2571, *L. monocytogenes* ATCC 51776, and *S. aureus* ATCC 25923). Moreover, the strain showed approximately 40% 2,2-diphenyl-1-picryl-hydrazyl (DPPH) radical- and 10% 2-azino-bis-(3-ethylbenzothiazoline-6-sulfonic acid (ABTS) radical-scavenging activity. In cell culture studies, human colon epithelial cells (Caco-2) were treated with LPLB5 (10^6^ and 10^7^ CFU/mL); the bacteria showed more than 70% adherence onto and a 32% invasion rate into the Caco-2 cells. LPLB5 significantly decreased the mRNA expression levels of pro-inflammatory cytokines (interleukin-1 beta (IL-1β), interleukin 6 (IL-6), and tumor necrosis factor-alpha (TNF-α)) and increased the mRNA expression levels of anti-inflammatory cytokines (interleukin-4 (IL-4), interleukin-10 (IL-10), and interferon-gamma (IFN-γ)) in lipopolysaccharide-stimulated Caco-2 cells. Our data suggest that LPLB5 is safe and possesses probiotic, antioxidant, and anti-inflammatory activities.

## 1. Introduction

Kimchi is one of the most important and broadly consumed traditional fermented vegetable-based foods in Korea [1]. There are almost 167 types of kimchi depending on the main ingredients, region, and manufacturing method [2]. Kimchi is fermented by many lactic acid bacteria (LAB), including the genera *Leuconostoc, Weissella*, and *Lactobacillus*. [3]. These LAB from kimchi have been reported that have desirable probiotic properties such as: (i) tolerance to acidity and bile (conditions found in the gastrointestinal (GI) tract), (ii) ability to adhere to intestinal epithelial cells, and (iii) potential for exerting health effects such as anti-inflammatory action, relief from diarrhea, anti-allergic effects, anti-cancer, anti-obesity effects, and cholesterol reduction [4,5,6,7,8,9]. 

Recently, nitrate-reducing LAB have been suggested as alternatives to decrease nitrite/nitrate usage in the meat industry [10]. Nitrate/nitrite are used as food additives in processed meat products as preservatives by inhibiting the growth of microorganisms including *Clostridium botulinum*, and as color fixatives [11]. However, along with a growing public concern about consuming chemical preservatives, there has been a considerable interest in natural alternatives such as vegetable-derived nitrate along with a nitrate-reducing starter culture [11]. Moreover, despite controversy regarding nitrate/nitrite consumption, nitric oxide (NO) from dietary-derived nitrate/nitrite have prophylactic and curative effects in colonic inflammation by increasing gastric mucosal blood flow and mucus formation [12,13]. Recent studies have reported that dietary nitrate/nitrite alleviate colitis by reducing inflammation, inhibiting apoptosis, and controlling colon microbiota via activation of the NO_3_^−^/NO_2_^−^/NO pathway. For example, nitrite supplementation alleviated dextran sodium sulfate (DSS)-induced colitis in mice by improving the colonic mucosal integrity, increasing epithelial cell wound healing, and maintaining the thickness of the colonic mucus layer [14]. Furthermore, the oral administration of dietary nitrate improved colon health and reduced apoptosis in colon epithelial cells [15]. Decreased inflammatory cell infiltration in the peripheral blood and colon was also observed in this past study. The bioactive NO is derived from the sequential reduction of nitrates through NO_3_^−^/NO_2_^−^/NO pathway [16,17]. The reduction of nitrate to nitrite is the initial step for supplying the bioactive NO [18]. This conversion is conducted by nitrate-reducing bacteria inhabiting the gastrointestinal track for food-originated nitrate, and there are numerous other pathways in the body for further reduction of nitrite to bioactive NO [19,20,21].

In this study, we aimed to isolate a new nitrate-reducing probiotic strain from kimchi. Subsequently, the strain was identified and its (1) safety properties, (2) probiotic characteristics, and (3) anti-inflammatory activity in the human colon epithelial cells (Caco-2) were evaluated.

## 2. Materials and Methods

### 2.1. Isolation of Nitrate-Reducing Bacterial Strains and Culture Condition

Nitrate-rich, traditionally fermented vegetable-based kimchi products including cabbage kimchi (*n* = 30), spinach kimchi (*n* = 2), leaf mustard kimchi (*n* = 10), turnip kimchi (*n* = 10), young radish kimchi (*n* = 10), radish water kimchi (*n* = 10), and cubed radish kimchi (*n* = 8) were purchased from a local market in Gwangju, South Korea. LAB was isolated from 10 g or 10 mL of each sample in a sterile stomacher bag. This bag contains 90 mL of sterile 0.85% (*w/v*) sodium chloride (NaCl) solution supplemented added with 0.01% (*v/v*) Tween 80. The sample was mixed in a stomacher (Seward Laboratory Systems Inc., Bohemia, NY, USA) for 5 min. After 10-fold serial dilutions, 0.1 mL of each dilution were spread onto De Man, Rogosa, and Sharpe (MRS) (Difco^TM^, BD Biosciences, Sparks, MD, USA) agar supplemented with sodium nitrate (NaNO_3_) (200 ppm) and cultured anaerobically for 48 h at 30 °C. Eight to twelve colonies per sample were isolated and incubated in an MRS broth for 48 h at 30 °C. Finally, the isolates were stored in MRS broth containing 20% (*v/v*) glycerol at −70 °C. 

### 2.2. Selection of Nitrate-Reducing Microorganism

To detect nitrate-reducing bacteria, the nitrate-tolerant isolates from the various kimchi types and one hundred strains of kimchi LAB (MGB0001–MGB0100) obtained from Microorganism and Gene Bank (MGB) were cultured in MRS broth containing 200 ppm NaNO_3_ at 30 °C for 48 h. Bacterial growth was measured by reading the optical density at 600 nm. After centrifugation (8000× *g*, 15 min, 4 °C), nitrate levels in the culture supernatant were determined using a LAQUA twin nitrate meter (Horiba, Kyoto, Japan). 

### 2.3. Microorganism Identification

A preliminary identification of the selected isolate was conducted by microscopic observation after Gram staining. Analytical Profile Index (API 50 CHL) strips (bioMérieux Co., Marcy l’Etoile, France) were used for determination of the carbohydrate-fermentation patterns of the selected isolate. To amplify a partial 16S rRNA fragment from the isolate, PCR was performed (Minicycler, MJ Research Inc., Waltham, MA, USA) using universal primers (27F: 5′–AGAGTTTGATCATGGCTCAG–3′ and 1492R: 5′–GGATACCTTGTTACGACTT–3′) [22]. To prepare PCR samples, bacterial DNA was extracted by centrifuging a 10 mL culture aliquot of the selected strain at 8000× *g* for 15 min. The resulting pellet was rinsed with sterile phosphate-buffered saline (PBS, pH 7.0) and resuspended in 1 mL of Tris- Ethylenediaminetetraacetic acid (EDTA) buffer (10 mM Tris, 5 mM EDTA, pH 8.0). Lysozyme (50 mg/mL; Sigma Chemical Co., St. Louis, MO, USA) was added to lysis the bacterial cells. Subsequently, DNA was extracted using the DNeasy tissue kit (QIAGEN, Valencia, CA, USA). The concentration and purity of the DNA were determined using a biophotometer (Eppendorf, Hamburg, Germany). The PCR conditions included an initial denaturation at 95 °C for 5 min, followed by 35 cycles of 45 s at 94 °C, 45 s at 52 °C, 1 min at 72 °C, and a final extension at 72 °C for 5 min. The PCR products were then ligated into a T vector (Invitrogen, Carlsbad, CA, USA). The sequencing of the 16S rRNA gene was conducted using a genetic analyzer (ABI Prism 3730 xl system, Applied Biosystems, Foster, CA, USA). The 16S rRNA gene sequences from the isolate were aligned with NCBI database (http://www.ncbi.nlm.nih.gov) using the basic local alignment search tool (BLAST) program (http://blast.ncbi.nlm.nih.gov/Blast.cgi). Multiple sequence alignments were assessed with the CLUSTAL_W program [23] and the BioEdit program was used to exclude alignment positions with gaps and unidentified bases [24]. Molecular evolutionary genetics analysis (MEGA) software was used to assess the phylogenetic tree [25]. A phylogenetic tree was constructed using the neighborhood-joining and maximum-parsimony criterion and bootstrap values based on 1000 replications [26].

### 2.4. Tolerance of the LAB to Acid, Bile, and Heat

The stress tolerance (tolerance to acid, bile, and heat) of the strain was tested using the viable colony counts method, as described previously [27] with some modifications. Briefly, an overnight culture of *Lactiplantibacillus plantarum* LB5 (LPLB5) was diluted to obtain an initial density of 10^7^ CFU/mL in MRS broth. Prior to inoculation, the pH of the MRS broth for the acid tolerance test was adjusted to either 2.5 or 6.5 (control) with 1 N HCl. To assess bile salt tolerance, the MRS broth was supplemented with or without (control) 0.3% (*w/v*) bile salt oxgall (Oxoid, Hampshire, UK). To evaluate the thermotolerance of the strain, the inoculated MRS broth (10^7^ CFU/mL) was cultured at 40, 50, and 60 °C for 1 h. To test the tolerance to acid and bile, the inoculated MRS broths (10^7^ CFU/mL) were incubated at 37 °C for 4 h and 24 h, respectively. After incubation, the remaining cells were counted using the spread plate method on MRS agar. For the acid and bile tolerance test, the survivability was calculated using following equation:

Survivability (%) = (Treatment CFU/mL)/(Control CFU/mL) × 100

### 2.5. Preparation of Pathogenic Bacteria and Antibacterial Activity

The antibacterial activity of LPLB5 was determined by the agar well diffusion assay as described previously by Varadaraj et al. [28]. Following indicator strains were used: *E. coli* O157:H7 ATCC 35150, *Pseudomonas aeruginosa* KCCM 12539, *Listeria monocytogenes* KCCM 40,307, and *Staphylococcus aureus* ATCC 25923. All these strains were cultured in tryptic soy broth at 37 °C for 48 h. In parallel, an overnight culture of the test strain LPLB5 in MRS medium was prepared, centrifuged (8000× *g*, 30 min, 4 °C), and the supernatant was used as antagonistic substance. A total of 100 μL of each pathogenic bacterium were spread on tryptic soy agar (TSA) (Difco^TM^, BD Biosciences, Sparks, MD, USA) plates. Then, 120 μL of cell-free supernatant were added to the wells on the agar. After an incubation at 37 °C for 24 h, the diameter of each clear zone of growth inhibition was measured in millimeters to determine the antagonistic effects. 

### 2.6. Antibiotic Susceptibility of the LAB Strain

The antibiotic susceptibility test was determined as described previously [29,30]. Antibiotic resistance to 13 antibiotics (KisanBio, Seoul, Korea): Ampicillin (10 µg), chloramphenicol (30 µg), clindamycin (10 µg), ciprofloxacin (5 µg), gentamicin (10 µg), doxycycline (30 µg), erythromycin (15 µg), kanamycin (30 µg), penicillin G (10 IU), streptomycin (10 µg), trimethoprim-sulfamethoxazole (25 µg), tetracycline (30 µg), and vancomycin (30 µg) were evaluated by the disc diffusion method. A total of 100 µL of LPLB5 (10^7^ CFU/mL) was spread onto MRS agar and each antibiotic disc was placed on the plate. After incubation (at 37 °C, 24 h), the diameter of the growth inhibition zones around each disk was measured. The results were evaluated according to the Clinical and Laboratory Standards Institute (CLSI) 2012 standards [31]. 

### 2.7. Enzyme Production by the LAB Strain

Enzyme production by the LPLB5 strain was measured using an API ZYM kit (BioMerieux, Lyon, France). The bacterial cells were harvested and resuspended in 0.85% NaCl to a concentration of 10^7^ CFU/mL. The samples were inoculated in API ZYM and incubated at 37 °C for 4 h. Then, reagents A and B were added for color development.

### 2.8. Hemolytic Activity

The hemolytic activity of LPLB5 was investigated using Colombia agar plates (Oxoid) containing 7% (*v/v*) sheep blood (Oxoid). LPLB5 was cultured at 37 °C for 24 h. A clear and colorless zone around the colonies showed β-hemolytic activity, formation of a green zone indicated α–hemolytic activity, and no change in the medium indicated γ-hemolytic activity [32].

### 2.9. Auto-Aggregation and Co-Aggregation

Auto- and co-aggregation were determined as described by Angmo et al. [33], with some modifications. For auto-aggregation, bacteria from an overnight culture were washed thrice and resuspended in PBS (Lonza, Walkersville, MD, USA) to yield a density of 10^7^ CFU/mL. The suspension was vortexed for 10 s and the absorbance at 600 nm was measured before incubation at 37 °C for 4 h. The absorbance of the upper phase was measured again after incubation. The auto-aggregation percentage was calculated using the following equation:

Auto-aggregation (%) = [1 − A_4h_/A_0h_] × 100

For the co-aggregation assay, equal volumes (2mL) of cell suspensions of LPLB5 and different pathogens (*E. coli* O157: H7 ATCC 35150, *E. coli* KCTC 2571, *L. monocytogenes* ATCC 51776, and *S. aureus* ATCC 25923) were vortexed for 10 s and incubated at 37 °C for 4 h. The absorbance of each mixed suspension was compared with those of the control tubes containing the LPLB5 (A_strain_) and the pathogen (A_pathogen_). The co-aggregation percentage was calculated using the following equation:

Co-aggregation (%) = [1 − A_mix_/(A_strain_ + A_pathogen_)/2] × 100

### 2.10. Antioxidant Activity of the LAB Strain

The 2,2-diphenyl-1-picryl-hydrazyl (DPPH) and 2-azino-bis-(3-ethylbenzothiazoline-6-sulfonic acid (ABTS) radical-scavenging activities of LPLB5 and *L. plantarum* ATCC 8014 were determined as described previously [34,35]. A total of 0.5 mL of DPPH reagent (0.1 mM) was mixed with an equal volume of bacterial suspension (10^6^ and 10^7^ CFU/mL), followed by incubation at 37 °C in the dark for 30 min. DPPH reagent added to ethanol served as a control. After centrifugation (14,000× *g*, 1 min, 4 °C), the absorbance of the samples was measured at 517 nm. 

ABTS reagent (14.8 mM) was mixed with 5 mM potassium persulfate (1:1, *v/v*) and left in the dark for 16 h at 25 °C. The ABTS solution was diluted with distilled water to obtain an absorbance of 0.700 ± 0.05 at 734 nm before use. A total of 900 µL of ABTS solution and 100µL of bacterial suspension (10^6^ and 10^7^ CFU/mL) were mixed and incubated at 25 °C in the dark for 15 min. ABTS solution added in ethanol was served as a control. After centrifugation at 14,000× *g* for 1 min at 4 °C, the absorbance of the samples was measured at 734 nm. The radical scavenging activity was calculated as follows:

Radical scavenging activity (%) = [1 − (A_sample_/A_control_) × 100]

### 2.11. Cell Culture and Treatments

The human colon carcinoma cell line was supplied by the Korean Cell Line Bank (Jongno-gu, Seoul, Korea) and cultured in Dulbecco’s modified Eagle’s medium (DMEM) (Welgene Inc., Gyeongsan-si, Korea) supplemented with 10% fetal bovine serum (FBS) (Welgene Inc., Gyeongsan-si, Korea) and 1% penicillin/streptomycin (*v/v*) at 37 °C in a humidified atmosphere containing 5% CO_2_. The overnight cultures of LPLB5 and *L. plantarum* ATCC 8014 were centrifuged, washed thrice with PBS, and resuspended in antibiotic-free DMEM to the desired concentration before treatment. 

### 2.12. Cytotoxicity of the LAB in Caco-2 Cells

The cytotoxicity of LPLB5 and *L. plantarum* ATCC 8014 were investigated using a lactate dehydrogenase (LDH) Cytotoxicity Assay Kit (Promega, Madison, WI, USA). Briefly, Caco-2 cells grown in 96-well plates were treated with different concentrations (10^6^, 10^7^, 10^8^, and 10^9^ CFU/mL) of LPLB5 and *L. plantarum* ATCC 8014 and incubated at 37 °C for 24 h. As a positive control, cells were treated with cell membrane lysis buffer 45 min before the end of incubation. Following this, the medium was transferred to a 1.5 mL tube and centrifuged to collect the supernatants. A total of 50 µL of the supernatant was mixed with 50 µL of CytoTox 96^®^ reagent, followed by incubation in the dark for 30 min. The absorbance was measured at 490 nm and the percentage of LDH release (%) was calculated as follows:

Cytotoxicity (%) = [(A_sample_ LDH release/A_maximum_ LDH release) × 100]

where A_maximum_ is the absorbance of the medium of the positive control (the medium of plates treated with cell membrane lysis buffer).

### 2.13. Adhesion and Invasion of the LAB Strain in Caco-2 Cells

The abilities of LPLB5 and *L. plantarum* ATCC 8014 to adhere to and invade Caco-2 cells was determined according to a previously described method [36] with some modifications. Overnight cultures of LPLB5 and *L. plantarum* ATCC 8014 were centrifuged, washed thrice with PBS, and resuspended in antibiotic-free DMEM at concentrations of 1 × 10^6^ CFU/mL and 1 × 10^7^ CFU/mL. The bacterial suspensions were added to the Caco-2 cell monolayer, followed by incubation for 2 h at 37 °C. After treatment, the cell monolayer was rinsed thrice with PBS to remove any non-adherent bacteria. Subsequently, the cells were detached with 200 µL of trypsin and lysed with 800 µL of 0.1% Triton X-100. Cell lysates containing adhered bacteria were serially diluted, spread on MRS agar, and incubated at 37 °C for 24 h.

For the invasion assay, Caco-2 cells were treated with LPLB5 cells similar to the case for the adhesion assay. After 2 h of incubation, the Caco-2 cells were washed with PBS, and fresh DMEM containing 100 µg/mL of gentamicin was added. A further 2 h of incubation was performed to kill the extracellular bacteria. At the end of the incubation, the cells were washed, detached, and lysed, and the viable colony counts were enumerated by the plate counting method.

### 2.14. Determination of the Anti-Inflammatory Activity in Caco-2 Cells

To evaluate the gene expression of pro-inflammatory (tumor necrosis factor-alpha (TNF-α), interleukin-1 beta (IL-1β), and interleukin 6 (IL-6)) and anti-inflammatory cytokines (interleukin-4 (IL-4), interleukin-10 (IL-10), and interferon-gamma (IFN-γ)), cells were grown in a 6-well plate at a concentration of 1 × 10^6^ cells/mL and treated with 10^6^ or 10^7^ CFU/mL of LPLB5 in antibiotic-free DMEM medium for 6 h. Next, the medium containing bacteria was withdrawn from plates and cells were challenged with lipopolysaccharide (LPS) (1 µL/mL) for another 2 h to initiate inflammatory responses and stimulate the release of inflammatory cytokines. After incubation, cells were rinsed thrice with PBS before RNA extraction. Total RNAs were extracted using TRIzol reagent and the TOPscript RT DryMIX kit (Enzynomics, Daejeon, Korea) and used for cDNA synthesis. To determine the mRNA expression levels, quantitative real-time PCR was carried out using a real-time PCR mix (SolGent, Daejeon, Korea) and the Roche LightCycler^®^ 96 System (Roche, Basel, Switzerland). The conditions used for PCR were as follows: Initial denaturation at 95 °C for 15 min, followed by 40 cycles at 95 °C for 10 s, 60 °C for 10 s, and 72 °C for 10 s. The reference gene glyceraldehyde 3-phosphate dehydrogenase (GAPDH) was used to quantify the relative mRNA expression levels. The sequences of the primers used for the target genes are shown in Table 1.

### 2.15. Statistical Analysis

Experiments were conducted in at least triplicate and the data are represented as the mean ± standard error of the mean (SEM). Statistical significance was analyzed using the SPSS-PASW statistic software version 18.0 Windows (SPSS, Chicago, IL, USA). Differences were considered statistically significant when *p*-values were < 0.05.

## 3. Results

### 3.1. Isolation and Selection of Nitrate-Reducing Microorganism

**Figure 1:**
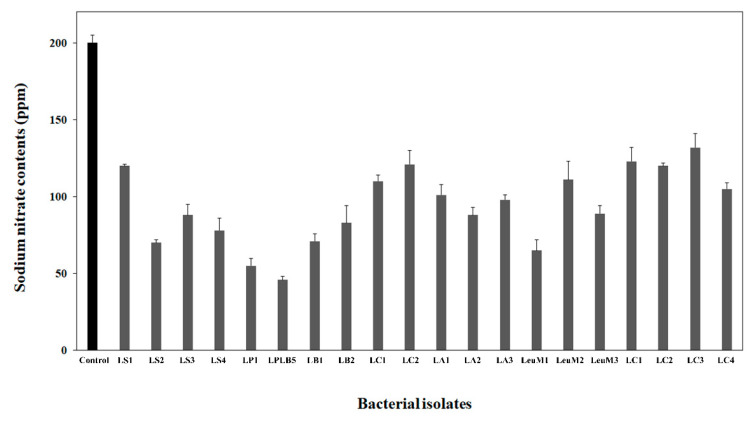
Nitrate reduction in the culture supernatant of bacterial isolates from kimchi. Nitrate contents in the culture supernatant were estimated using a nitrate meter. *Lactiplantibacillus plantarum* LB5 (LPLB5) showed the highest nitrate reducing ability. Control, De Man, Rogosa, and Sharpe (MRS) broth containing sodium nitrate; LS, *Lb. sakei*; LP, *L. plantarum*; LB, *Le. brevis*; LC, *Lb. curvatus*; LA, *Lb. alimentarius*; LeuM, *Leu. mesenteroides*; and LeuC, *Leu. citreum.*

Eight hundred isolates of nitrate-tolerant microorganisms were isolated from different types of kimchi containing high nitrate concentrations. The isolates and kimchi LAB obtained from MGB were cultured in MRS broth supplemented with NaNO_3_, followed by a screening assay for nitrate reduction. Twenty microorganisms related to kimchi fermentation including *Lactobacillus sakei* (four strains), *Levilactobacillus brevis* (two strains), *Lactiplantibacillus plantarum* (two strains), *Lb. alimentarius* (three strains), *Lb. curvatus* (two strains), *Leuconostoc mesenteroides* (three strains), and *Leu. citreum* (four strains) with excellent nitrate reduction potential were selected. Measuring the nitrate content in the supernatant of the different cultures, isolate LPLB5 was selected as the strain with excellent nitrate reduction capabilities (Figure 1).

**Figure 2:**
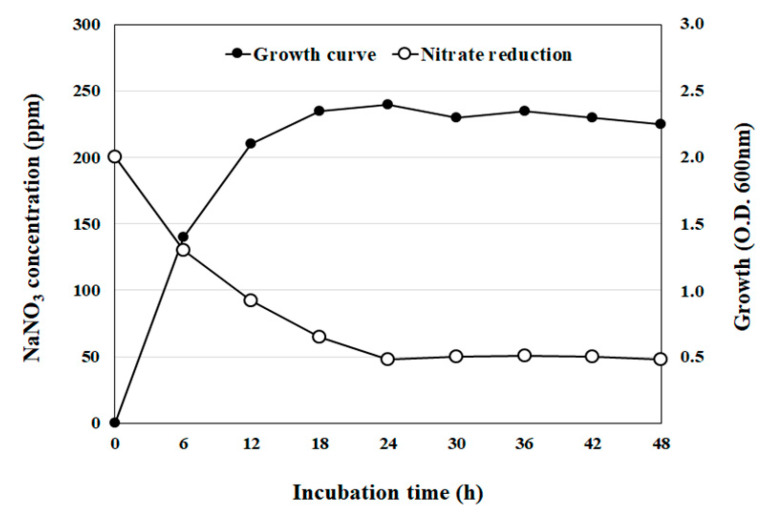
Bacterial growth curve and nitrate reduction by isolate LPLB5. The cells were grown at 30 °C in an MRS broth containing 200 ppm sodium nitrate (NaNO_3_). Cell growth was determined by measuring the optical density at 600 nm. Nitrate levels in the culture supernatant were determined using a nitrate meter.

For this strain, it was observed that the initial nitrate concentration of 200 ppm decreased rapidly within the first 24 h and remained constant at 50 ppm for the next 24 h of cultivation. Furthermore, rapid growth could be observed within the first 12 h of incubation and the stationary phase was reached after 18 h (Figure 2).

### 3.2. Identification of LPLB5

**Figure 3:**
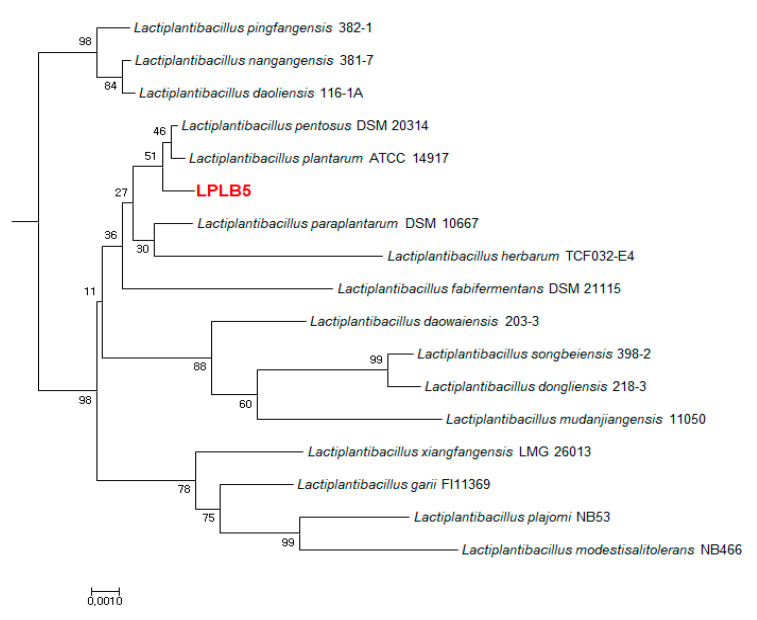
Neighbor-joining phylogenetic tree constructed based on 16S rRNA gene sequencing. The position of strain LPLB5 and its closest members of the genus *Lactiplantibacillus* were shown. Bootstrap percentages (≥30%) based on 1000 replications are given at the branch points. Bar, 0.01 substitutions per nucleotide position.

The selected LPLB5 strain was Gram-positive and utilized 99% of sugars which is known to be used by *Lactobacillus* species in biochemical tests using API 50 CHL. Gas-free acid was weakly produced in CHL medium with the following carbohydrates from the API 50CH gallery: Ribose, glucose, fructose, maltose, melibiose, sucrose, raffinose, gluconate, and 5-ketogluconate (data not shown). BLASTn tool was used to compare the 16S rRNA gene sequence of LPLB5 to the NCBI database. Phylogenetic relationships of novel bacterial species were facilitated by using 16S rRNA gene sequence. LPLB5, with its high nitrate reduction activity, was identified as a strain of *Lactiplantibacillus plantarum* (98% sequence homology) upon 16S rRNA sequencing and was designated as *Lactiplantibacillus plantarum* (LP) LB5 (Figure 3).

### 3.3. Effects of Acid, Bile Salts, and Heat Tolerance on the Survival of LPLB5

**Figure 4:**
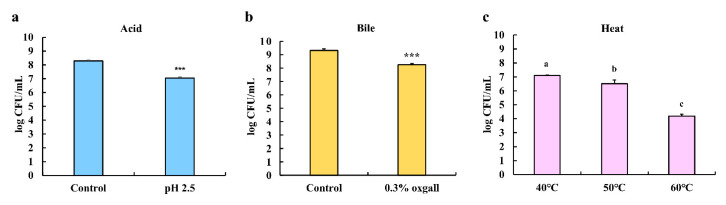
Acid, bile salt, and heat tolerance of LPLB5. The viable cells were counted after exposure to (**a**) pH 6.5 (control) or 2.5 for 4 h (**b**) without (control) or with 0.3% bile salt (oxgall) for 24 h and (**c**) 40, 50, and 60 °C for 1 h. The results represent the mean ± SEM, with *n* = 3. *** (*p* < 0.001) indicates a significant difference vs. control. The different letters on the error bars indicate statistically significant differences between the groups (*p* < 0.05).

The tolerance of LPLB5 to various challenges (acidity, bile, and heat) was determined by measuring viable cell counts after each challenge. Compared to the control (8.29 ± 0.06 log CFU/mL. pH 6.5), LPLB5 showed a relatively high viability (7.04 ± 0.07 log CFU/mL) after 4 h in an acidic medium (pH 2.5) as shown in Figure 4a. The bile tolerance was examined after growing LPLB5 in a medium containing 0.3% of the bile salt oxgall for 24 h (Figure 4b). Compared to the control (9.31 ± 0.86 log CFU/mL, without bile salt), a relatively high viability (8.24 ± 0.31 log CFU/mL) was also observed. Finally, the resistance of LPLB5 to heat challenges was investigated by culturing it at 40, 50, and 60 °C for 1 h (Figure 4c). The viable count of LPLB5 cultured at 40 °C was 7.1 log CFU/mL; its survival decreased significantly at higher temperatures (50 and 60 °C).

### 3.4. Antibacterial Activity of LPLB5

**Figure 5:**
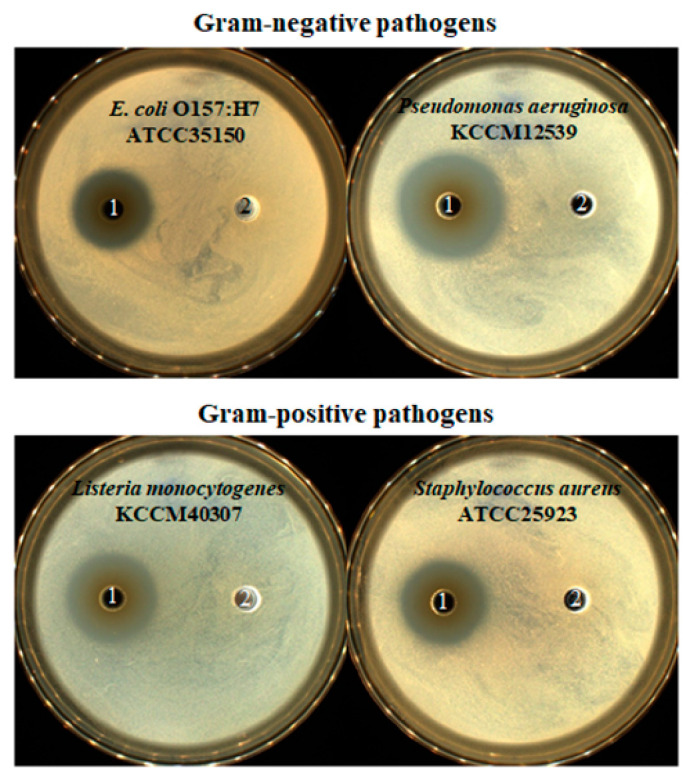
Antibacterial activity of LPLB5. 1, culture supernatant of LPLB5; 2, MRS broth as control. Antibacterial activity of culture supernatant of LPLB5 against *E. coli* O157:H7, *p. aeruginosa, Leu. monocytogenes, S. aureus* using the agar well diffusion assay. Four pathogenic bacteria were spread on tryptic soy agar (TSA) plates, then 120 μL of culture supernatant was added to the wells, previously punched into the agar. The plates were incubated at 37 °C for 24 h.

The culture supernatant of LPLB5 was tested for its antibacterial activity against *E. coli* O157:H7 *Pseudomonas aeruginosa*, *Listeria monocytogenes,* and *Staphylococcus aureus*, which are Gram-negative and Gram-positive foodborne pathogens, respectively. Results showed that each of these strains tested was inhibited differently (Figure 5). No antibacterial activity was observed in the presence of the control MRS liquid medium. Thus, the culture supernatant of LPLB5 was found to have an antibacterial activity against representatives of Gram-negative and Gram-positive food poisoning bacteria. Thus, LPLB5 was classified among the LAB with excellent antibacterial activity against representatives of pathogenic bacteria.

### 3.5. Antibiotic Susceptibility of LPLB5

LPLB5 was sensitive to eight antibiotics (ampicillin, chloramphenicol, clindamycin, doxycycline, erythromycin, penicillin G, trimethoprim-sulfamethoxazole, and tetracycline), had an intermediate resistance to two antibiotics (ciprofloxacin and gentamicin), and was resistant to three antibiotics (kanamycin, streptomycin, and vancomycin) (Table 2).

### 3.6. Enzyme Activities of LPLB5

A β-glucuronidase activity was absent in LPLB5 (Table 3). In contrast, LPLB5 showed high levels of leucine arylamidase, α-galactosidase, and β-galactosidase activity.

### 3.7. β-hemolytic Activity of LPLB5

LPLB5 was tested for hemolytic activity as a potential probiotic strain. A γ-hemolysis was observed while β-hemolysis was not detected in LPLB5 (Figure S1).

### 3.8. Auto-Aggregation and Co-Aggregation Ability of LPLB5

**Figure 6:**
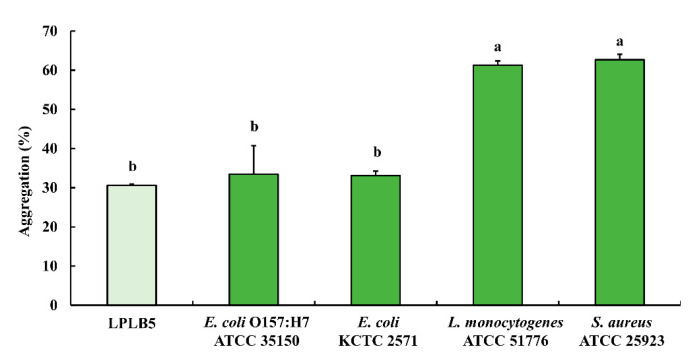
Auto- and co-aggregation ability of LPLB5. The absorbance of the bacterial suspension at was measured at 600 nm and that of the upper phase was measured at 600 nm again after incubation at 37 °C for 4 h. The results represent the mean ± SEM, with *n* = 3. The different letters on the error bars indicate statistically significant differences between the groups (*p* < 0.05).

The results of the auto- and co-aggregation assays are shown in Figure 6. The auto-aggregation of LPLB5 was about 30.61%. LPLB5 exhibited co-aggregation ability with four pathogens (33.45% with *E. coli* O157: H7 ATCC 35150, 33.08% with *E. coli* KCTC 2571, 61.30% with *L. monocytogenes* ATCC 51776, and 62.69% with *S. aureus* ATCC 25923).

### 3.9. Antioxidant Activity of LPLB5

**Figure 7:**
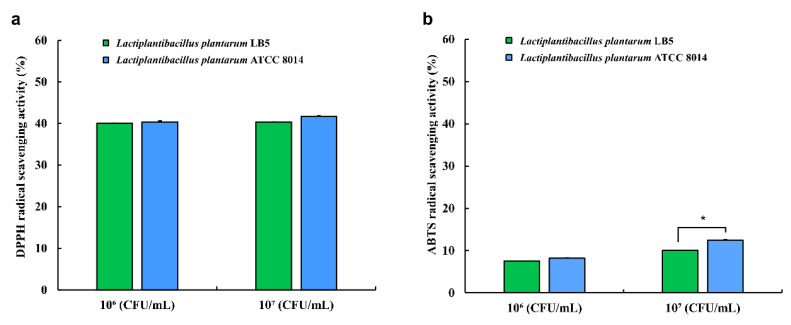
Antioxidant activity of LPLB5 and *L. plantarum* ATCC 8014. (**a**) 2,2-diphenyl-1-picryl-hydrazyl (DPPH) and (**b**) 2-azino-bis-(3-ethylbenzothiazoline-6-sulfonic acid (ABTS) radical scavenging activities were determined. The results represent the mean ± SEM, with *n* = 3. * indicates a significant difference vs. control (* *p* < 0.05).

To evaluate the antioxidant capacity of LPLB5, DPPH radical- and ABTS radical-scavenging analyses were performed (Figure 7). The results showed that LPLB5 had DPPH radical-scavenging activity of 40%; there was no significant difference between the DPPH radical-scavenging activities with 10^6^ and 10^7^ CFU/mL of LPLB5 (Figure 7a). An established probiotic strain, *L. plantarum* ATCC 8014, showed a DPPH radical scavenging activity similar to that of LPLB5. At 10^7^ CFU/mL, both LPLB5 and *L. plantarum* ATCC 8014 had ABTS radical-scavenging activities of about 10% (Figure 7b), with that of *L. plantarum* ATCC 8014 being significantly higher.

### 3.10. Cytotoxicity of LPLB5 in Caco-2 Cells

**Figure 8:**
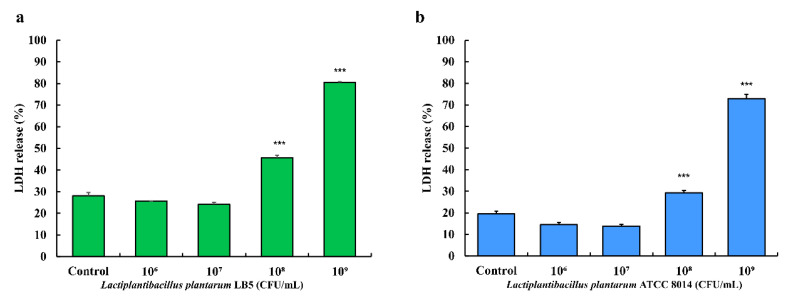
Effects of (**a**) LPLB5 and (**b**) *L. plantarum* ATCC 8014 on lactate dehydrogenase (LDH) release from human colon epithelial cells (Caco-2) cells. The Caco-2 cells were treated with LPLB5 or *L. plantarum* ATCC 8014 (10^6^, 10^7^, 10^8^, and 10^9^ CFU/mL) for 24 h. The results represent the mean ± SEM, with *n* = 3. *** indicates a significant difference vs. control (*** *p* < 0.001).

Compared to the control (no treatment with LPLB5), Caco-2 cells treated with a concentration of 10^8^ and 10^9^ CFU/mL of LPLB5 showed significantly higher LDH release (Figure 8a). Similarly, the LDH release increased in cells treated with 10^8^ and 10^9^ CFU/mL of *L. plantarum* ATCC 8014 (Figure 8b). As a result of the LDH assay, the concentration of 10^6^ and 10^7^ CFU/mL were selected for further cell culture studies.

### 3.11. Adhesion and Invasion of LPLB5 to Caco-2 Cells

**Figure 9:**
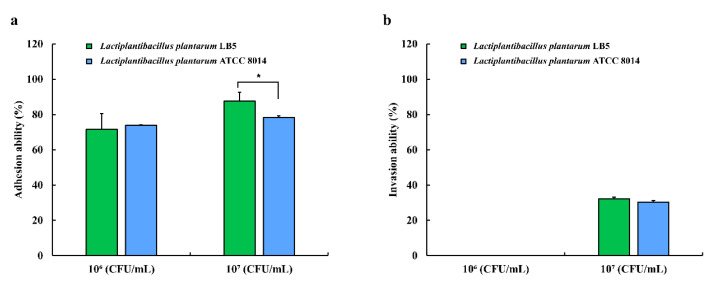
(**a**) adhesion and (**b**) invasion of LPLB5 and *L. plantarum* ATCC 8014 in Caco-2 cells. The Caco-2 cell were treated with LPLB5 or *L. plantarum* ATCC 8014 (10^6^ and 10^7^ CFU/mL) for 2 h at 37 °C. For the invasion assay, the cells were then incubated for a further 2 h with gentamicin. The viable cell counts were enumerated before and after incubation. Results represent the mean ± SEM, with *n* = 3. * indicates a significant difference vs. control (* *p* < 0.05).

The adhesion ability of LPLB5 cells was evaluated by incubating a Caco-2 cell monolayer with LPLB5 cells (10^6^ and 10^7^ CFU/mL) for 2 h. For comparison, identical concentrations of *L. plantarum* ATCC 8014 were also used as controls. The adhesion rates for LPLB5 and *L. plantarum* ATCC 8014 were more than 70% at cell densities of 10^6^ and 10^7^ CFU/mL (Figure 9a). There was no significant difference between the adhesion rates of LPLB5 (71.60%) and *L. plantarum* ATCC 8014 (74.0%) at a cell density of 10^6^ CFU/mL. However, the adhesion rate of LPLB5 (87.66%) was significantly higher than that of *L. plantarum* ATCC 8014 (78.26%) at a cell density of 10^7^ CFU/mL (Figure 9a). Invasion into Caco-2 cells was not observed at a cell density of 10^6^ CFU/mL for both, LPLB5 and *L. plantarum* ATCC 8014, whereas the invasion rates were 32.28% (LPLB5) and 30.34% (*L. plantarum* ATCC 8014) at a cell density of 10^7^ CFU/mL (Figure 9b).

### 3.12. Anti-Inflammatory Activity of LPLB5 in Caco-2 Cells

**Figure 10:**
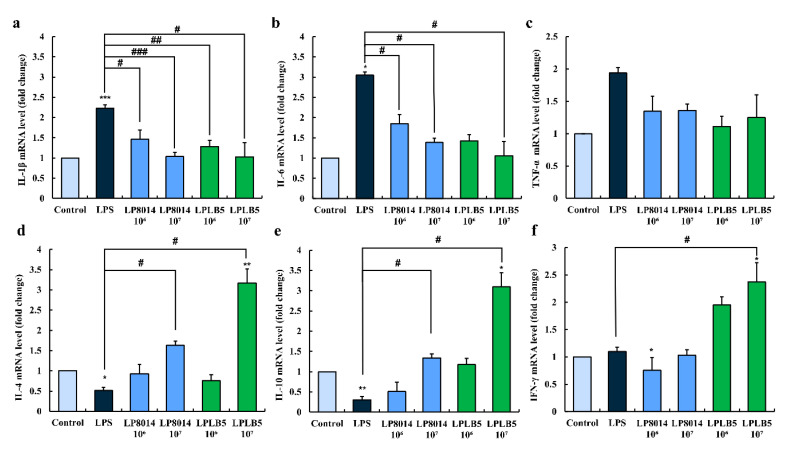
Effects of LPLB5 and *L. plantarum* ATCC 8014 (LP8014) on the mRNA expression levels of (**a**–**c**) pro-inflammatory cytokines (IL-1β, IL-6, and TNF-α) and (**d**–**f**) anti-inflammatory cytokines (IL-4, IL-10, and IFN-γ) in LPS-stimulated Caco-2 cells. Cells were pretreated with Dulbecco’s modified Eagle medium (DMEM), LPLB5, or *L. plantarum* ATCC 8014 (10^6^ and 10^7^ CFU/mL) for 6 h and then stimulated with LPS for 2 h. The results represent the mean ± SEM, with *n* = 3. * indicates a significant difference vs. control (* *p* < 0.05, ** *p* < 0.01, *** *p* < 0.001) and # indicates a significant difference vs. LPS-treated group (# *p* < 0.05, ## *p* < 0.01, ### *p* < 0.001).

The mRNA levels of three pro-inflammatory cytokines increased following LPS challenge (Figure 10a–c). However, the levels of IL-1β and IL-6 were significantly decreased when the cells were pretreated with 10^6^ and 10^7^ CFU/mL of LPLB5 or *L. plantarum* ATCC 8014 (Figure 10a,b). In addition, the expression levels of TNF-α showed a decreasing trend in response to the pretreatment with LPLB5 and *L. plantarum* ATCC 8014 (Figure 10c). The mRNA levels of anti-inflammatory cytokines IL-4, IL-10, and IFN-γ were upregulated in LPS-treated Caco-2 cells pretreated with 10^7^ CFU/mL of LPLB5 (Figure 10d–f).

## 4. Discussion

Bacterial nitrate reduction is important to yield nitrite and NO, which plays an important role as colorants and antimicrobial agents in cured meats [18]. With increasing consumer interest for natural, organic, and clean-label foods, nitrate-reducing bacteria have been used to substitute synthetic nitrite in cured meats [37]. Besides, it has been reported that bacterial nitrate reduction has beneficial effects on host health in various ways. In previous studies, nitrite produced by bacterial nitrate reduction is an important storage pool for NO in blood and tissues when nitric oxide synthase-mediated NO production is not sufficient [38,39,40]. Moreover, nitrite may act as a bactericidal agent against enteropathogens in the gut when nitrite is combined with acids [41]. Moreover, NO produced through the nitrate-nitrite-nitric oxide reductive pathway has been reported to have prophylactic and therapeutic effects in colon inflammation by improving gastric mucosal blood flow and mucus formation [12,13,21]. To generate NO and exert such effects, nitrate should be first reduced into nitrite [18]. In this study, we isolated 800 LAB from kimchi and selected one strain (LPLB5) with an excellent nitrate reduction capability. This strain was identified as *Lactiplantibacillus plantarum* by 16s rRNA gene sequencing and its safety and probiotic properties were evaluated.

Probiotics are expected to survive under human GI conditions such as acidic pHs and the presence of bile to be able to exert health benefits on the host [33]. The pH of gastric juice is usually 3.0, and thus, a pH of 2.0 is normally used in in vitro studies to simulate gastric conditions [42]. In our study, a pH of 2.5 was selected considering the possibility that the probiotic strain will be buffered by food matrices [43]. LPLB5 was exposed to 0.3% bile salt (oxgall) for 24 h, which is the common bile salt concentration in the human GI tract [44]. Compared to the control, our data showed that 84.91% and 92.98% of LPLB5 cells survived in media treated with acid and bile salt, respectively. Similar to our results, *L. plantarum* CP134 isolated from kimchi exhibited a 98.42% survival rate after 2 h of incubation at pH 2.5 and a 95.65% survival rate after 8 h of incubation with 0.3% oxgall [27]. These data indicate that LPLB5 has good probiotic properties that are similar to other kimchi-derived probiotics. Thermotolerance is an important characteristic of a probiotic because probiotics with thermotolerance can survive during food processing. It has previously been shown that *L. plantarum* K8, another probiotic strain from kimchi, exhibited a high tolerance to a temperature of 70 °C for 80 s [45], and *Weissella* strains showed at least 60% survivability at 60 °C for 3 min [46]. In our study, LPLB5 showed a relatively high thermotolerance under severe temperature conditions such as 50 and 60 °C for 1 h. The survival rates were approximately 91.7% and 59%, respectively. Taken together, our results demonstrate that LPLB5 can be used as probiotic with regard to its resistance to acid, bile, and heat.

The antimicrobial activity is one of the most important properties of starters and probiotics. Our data showed that LPLB5 inhibited the growth of representatives of both Gram-negative (*E. coli* O157:H7 and *P. aeruginosa*) and Gram-positive (*L. monocytogenes* and *S. aureus*) food-borne bacteria. It has been shown that LAB, including some *L. plantarum* strains, have an antagonistic effect against pathogens by producing antimicrobial metabolites including organic acids, diacetyl, hydrogen peroxide, and peptides or proteins named bacteriocins [47,48,49]. Similarly, our data suggest that LPLB5 has an antimicrobial activity against pathogenic bacteria.

LAB have earned the status of “Generally regarded as safe” (GRAS) by the US Food and Drug Administration (FDA), because they have been consumed in various traditional fermented foods for a long time. However, it is necessary to evaluate their resistance to various antibiotics because of concerns about the transmission of antibiotic resistance genes to pathogenic bacteria [50]. The bacterial resistance to antibiotics is classified into intrinsic (the inherent ability to resist the activity of antimicrobials) and acquired (through acquisition of additional genes or spontaneous mutations in the bacterial genome) resistance [50,51]. It has been known that intrinsic resistance is not horizontally transferable and presents no risk in non-pathogenic bacteria, while acquired resistance, in particular that based on additional genes, might be transferred horizontally among bacteria [50,51]. *L. plantarum* strains are usually susceptible to cell wall synthesis inhibitors like β-lactam antibiotics (e.g., ampicillin and penicillin) except of oxacillin and cephalosporins but resistant to glycopeptides (e.g., vancomycin) [51]. *L. plantarum* strains are also susceptible to antibiotics inhibiting peptidoglycan synthesis, such as chloramphenicol, clindamycin, erythromycin, and tetracycline, but are resistant to aminoglycosides (e.g., gentamicin, kanamycin, and streptomycin) [52]. In addition, *L. plantarum* are susceptible to antibiotics inhibiting nucleic acid synthesis (e.g., ciprofloxycin and trimethoprim-sulfonamide) [53,54,55]. Similar to previous data, our results demonstrated that LPLB5 exhibited resistance to aminoglycosides (i.e., kanamycin and streptomycin) and glycopeptide antibiotics (i.e., vancomycin), which have been characterized as intrinsic for lactiplantilactobacilli [56,57]. Based on its susceptibility to the other antibiotics tested, LPLB5 appears to be safe and non-harmful to the host, in terms of antibiotic resistance.

To evaluate the safety of the probiotic strain, the production of harmful enzymes such as β-glucuronidase was investigated using the API ZYM kit. β-Glucuronidase is an enzyme that catalyzes the cleavage of the β-glucuronosyl-O-bonds and liberates xenobiotics, which can cause carcinogenesis in the colon [58]. According to our data, LPLB5 showed high levels of leucine arylamidase, α-galactosidase, and β-galactosidase, while β-glucuronidase activity was not detected. In a previous study, seven *Limosilactobacillus fermentum* strains isolated from Tulum cheese showed high levels of leucine arylamidase and β-galactosidase, and four strains exhibited high levels of α-galactosidase [59]. Leucine arylamidase has proteolytic activity by hydrolyzing the N-terminal L-leucine from peptides [60]. α-Galactosidase catalyzes the hydrolysis of raffinose family oligosaccharides present in leguminous foods which causes flatulence and intestinal discomfort [61]. Additionally, it is noteworthy that β-galactosidase can alleviate lactose intolerance by breaking down lactose into glucose and galactose [62]. Thus, our data indicate that LPLB5 is safe for probiotic use.

β-hemolysis is defined as a transparent zone surrounding bacterial colonies on blood agar and is a virulence factor for pathogens. Members of group A *Streptococcus* (GAS) and group B *Streptococcus* (GBS) produce the exotoxins streptolysin S and β-hemolysin/cytolysin, respectively [63]. By producing these hemolysins, pathogens are able to lyse red blood cells and cause severe disease in humans (e.g., pharyngitis, acute rheumatic fever, rheumatic heart diseases, acute glomerulonephritis, and invasive infections) [64]. Herein, LPLB5 showed a complete absence of β-hemolysis, indicating that it is safe and non-pathogenic.

Auto-aggregation, the aggregation of bacteria belonging to the same strain, is important for probiotics to tolerate the extreme conditions in the GI tract and to adhere to the intestinal epithelial cells [65]. Another type of aggregation is co-aggregation, whereby probiotics coagulate with pathogens. Through co-aggregation, bacteria can prevent colonization by pathogens and inhibit the growth of adjacent pathogens by producing antibacterial substances [66]. Our data showed that LPLB5 exhibited 30% auto-aggregation and 33–60% co-aggregation with four pathogens. A previous study showed that fifteen *L. plantarum* strains showed 24.16–41.39% auto-aggregation and 21.36–32.16% co-aggregation with *E. coli* O157: H7 after 5 h of incubation [67]. In another study, the auto-aggregation of *L. plantarum* strains (IFPL33, IFPL81, IFPL150, IFPL156, and IFPL162) after 6 h of incubation ranged from 15.23–38.14%, and their co-aggregation with *S. aureus* CECT240, *L. monocytogenes* CECT5672, and *E. coli* O157:H7 CECT5947 after 24 h of incubation was greater than 40% [68]. Compared to these previous data, LPLB5 seems to have a good auto-aggregation ability and a high co-aggregation capability, particularly with *L. monocytogenes* and *S. aureus*.

Previous studies have reported that probiotics can reduce oxidative stress by chelating metal ion, expressing their own anti-oxidases, generating antioxidant metabolites, increasing anti-oxidase and metabolite levels in the host, mediating antioxidant signaling pathways, and regulating the gut microbiota [69]. For example, *L. plantarum* ZDY2013 and *Bifidobacterium bifidum* WBIN03 from acid beans have been shown to exhibit a protective effect against H_2_O_2_-induced oxidative stress in HT-29 cells and DSS-induced colitis in mice [70]. In addition, the oral administration of *Li. fermentum* 5716 alleviated trinitrobenzene sulphonic (TNBS)-induced colitis in rats by increasing the glutathione (GSH) levels and decreasing TNF-α and NO production [71]. Our results showed that LPLB5 demonstrated about 40% and 10% DPPH and ABTS radical-scavenging activities, respectively. Such low ABTS radical-scavenging activities have also been reported in previous studies. For example, *Le. brevis* B13-2 and *Lacticaseibacillus rhamnosus* GG have been reported to have 19.98 and 23.08% ABTS radical-scavenging activities, respectively [35]. *Lactobacillus* strains isolated from infant feces showed up to 37% DPPH radical-scavenging activity [72]. In comparison to these previous reports, our data suggest that LPLB5 has strong antioxidant activity, particularly DPPH radical scavenging activity.

To determine the optimal concentration of bacterial cells in cell culture studies, a LDH assay was performed. The leakage of intracellular LDH into the culture medium is an indicator of cell death due to cell membrane damage [73]. For example, some enteropathogenic *E. coli* strains increase intestinal permeability by attaching cells and causing an intestinal barrier dysfunction [74]. It has also been suggested that a high concentration of *Lactobacillus* strains can damage the cell membrane integrity. Accordingly, the LDH level was increased when human colorectal adenocarcinoma cell line, HT-29 were treated with *Lb. delbrueckii* subsp. *bulgaricus* BCRC 10,696 (10^9^ CFU/mL) and *Li. reuteri* BCRC 14,625 (10^8^ CFU/mL) [75]. In addition, *Li. fermentum* AGR1487 reduced the intestinal barrier integrity by increasing the expression of tubulin genes and microtubule-related proteins involved in tight junction disassembly [76]. Similar to the previous studies, our data showed that Caco-2 cells treated with 10^8^ and 10^9^ CFU/mL of both LPLB5 and *L. plantarum* ATCC 8014 exhibited significantly higher LDH release than the control. Therefore, the cell densities of 10^6^ and 10^7^ CFU/mL were selected for further in vitro assays.

Adhesion of probiotics to the intestinal mucosa is closely related to their colonization in the GI tract. The adhesion properties of probiotics can be affected by various factors, including non-specific adhesion (e.g., hydrophobic and electrostatic interactions) and specific binding to molecules such as the mannose-specific adhesin (Msa) of *L. plantarum* WCFS1 [77,78]. Thus, evaluation of the adhesion ability of probiotics using cultured human epithelial cells is an initial step to identify whether they can colonize the gut. In a previous study, five *L. plantarum* strains isolated from Siahmazgi cheese showed 73.75%–89.29% adhesion to Caco-2 cells [79]. In addition, *L. plantarum* B282 isolated from olive exhibited 60% adhesion to Caco-2 cells [80]. In our study, 10^7^ CFU/mL LPLB5 showed an 87.7% adherence ability, which is higher than that of *L. plantarum* ATCC 8014. Based on previous studies, LPLB5 seems to have a good colonization ability, which is essential for potential probiotic microorganism. Pathogens such as *L. monocytogenes, Shigella* spp., *Salmonella* spp., *Yersinia* spp., and *E. coli* can invade epithelial cells and cause inflammation [81]. Therefore, the invasion ability of microorganisms is generally investigated to ensure the safety of probiotics. Our results demonstrated that LPLB5 exhibited a 32% invasion rate, which is similar to that of *L. plantarum* ATCC 8014. Therefore, our data indicate that LPLB5 is safe because LPLB5 and a well-established probiotic strain, *L. plantarum* ATCC 8014, showed identical invasion abilities, but only at a high concentration (10^7^ CFU/mL).

Probiotics adhere to intestinal epithelial cells and exert immunomodulatory effects by increasing peripheral immunoglobulin production and IgA secretion and modulating inflammatory cytokine production [82]. In a previous study, LPS-challenged Caco-2 cells pretreated with *Lb. gasseri* JMI showed decreased mRNA levels of pro-inflammatory cytokines (IL-1β, IL-6, IL-8, and TNF-α) and increased mRNA levels of anti-inflammatory cytokines (IL-4, IL-10, TGF-β3, and IFN-γ) [83]. In addition, it has been shown that *Lb. gasseri* JM1 exerts its immunomodulatory effects by activating the toll-like receptor 2 and nucleotide binding oligomerization domain containing protein 2-mediated phosphatidylinositol 3-kinase (PI3K)/protein kinase B (Akt) signaling pathway in Caco-2 cells [83]. In this study, pretreatment of Caco-2 cells with LPLB5 ameliorated LPS-induced inflammatory responses by downregulating the mRNA levels of pro-inflammatory cytokines (e.g., IL-1β, IL-6, and TNF-α) and upregulating the mRNA levels of anti-inflammatory (e.g., IL-4, IL-10, and IFN-γ) cytokines.

## 5. Conclusions

*Lactiplantibacillus plantarum* LPLB5, a nitrate-reducing bacterium isolated from kimchi, seems to be non-pathogenic and has a desirable nitrate removal ability. The probiotic potential of LPLB5 is evidenced by its good survivability to various challenges, auto- and co-aggregation, adhesion ability, as well as antioxidant and anti-inflammatory effects. Therefore, LPLB5 could be a good candidate probiotic strain that for application in food/pharmaceutical industries.

## Figures and Tables

**Table 1:** Primer sequences used in the current study.

Name of Genes	Primer Sequence (5′-3′)
IL-1β ^a^	(F) TGT ACC TGT CCT GCG TGT TGA AAG (R) CTG GGC AGA CTC AAA TTC CAG CTT
IL-6	(F) ACA GCC ACT CAC CTC TTC AGA AC (R) TTT TCT GCC AGT GCC TCT TTG C
TNF-α	(F) AAG CCC TGG TAT GAG CCC ATC TAT (R) AGG GCA ATG ATC CCA AAG TAG ACC
IL-4	(F) TCA TTT TCC CTC GGT TTC AG (R) AGA ACA GAG GGG GAA GCA GT
IL-10	(F) TCA GGG TGG CGA CTC TAT (R) TGG GCT TCT TCT AAA TCG TTC
IFN-γ	(F) ATA TCT TGG CTT TTC AGC TC (R) CTC CTT TTT CGC TTC CCT GT
GAPDH	(F) GAC CCC TTC ATT GAC CTC AAC TAC (R) ATG ACA AGC TTC CCG TTC TCA G

^a^ IL-1β, interleukin-1 beta; IL-6, interleukin 6; TNF-α, tumor necrosis factor-alpha; IL-4, interleukin-4; IL-10, interleukin-10; IFN-γ, interferon-gamma; and GAPDH, glyceraldehyde 3-phosphate dehydrogenase.

**Table 2:** Antibiotic susceptibility of LPLB5.

Antibiotic Discs	Zone Diameter (mm)	
	Mean ± SE ^a^	CLSI ^b^
Ampicillin, 10 µg	35.33 ± 0.98	S
Chloramphenicol, 30 µg	37.33 ± 0.98	S
Clindamycin, 10 µg	27.67 ± 1.19	S
Ciprofloxacin, 5 µg	14.33 ± 0.54	I
Gentamicin 10 µg	15.00 ± 0.00	I
Doxycycline, 30 µg	30.00 ± 2.45	S
Erythromycin, 15 µg	36.67 ± 0.72	S
Kanamycin, 30 µg	11.67 ± 0.27	R
Penicillin, 10 IU	38.00 ± 1.25	S
Sreptomycin, 10 µg	10.67 ± 0.27	R
Trimethoprim-sulfamethoxazole, 25 µg	27.33 ± 0.54	S
Tetracycline, 30 µg	24.33 ± 0.98	S
Vancomycin, 30 µg	- ^c^	R

^a^ Standard error; ^b^ CLSI, Clinical and Laboratory Standards Institute; ^c^ Indicates no inhibition zone. The inhibition zones are evaluated according to the standard values given by CLSI. Susceptible (S) ≥ 20, Intermediate (I) ≅ 15–19, Resistant (R) ≤ 14 (CLSI, 2012).

**Table 3:** Quantification of enzymatic activity of LPLB5 using the API ZYM kit.

Enzyme	*Lactiplantibacillus plantarum* LB5
Control	0 ^a^
Alkaline phosphatase	1
Esterase	2
Esterase lipase	2
Lipase	0
Leucine arylamidase	5
Valine arylamidase	1
Cystine arylamidase	1
Trypsin	1
α-Chymotrypsin	0
Acid phosphatase	2
Naphthol-AS-BI-phosphohydrolase	2
α-Galactosidase	5
β-Galactosidase	5
β-Glucuronidase	0
α-Glucosidase	3
β-Glucosidase	0
N-Acetyl-β-glucosaminidase	0
α-Mannosidase	0
α-Fucosidase	0

^a^ 0, 0 nmol; 1, 5 nmol; 2, 10 nmol; 3, 20 nmol; 4, 30 nmol; and 5, ≥40 nmol.

## Supplementary Materials

The following are available online at https://www.mdpi.com/2304-8158/9/12/1777/s1, Figure S1: Hemolytic activity of LPLB5.
