# Supplementary material for: Probiotic Properties of Lactiplantibacillus plantarum LB5 Isolated from Kimchi Based on Nitrate Reducing Capability
Source: Foods. 2020 Nov 30;9(12):1777. doi: 10.3390/foods9121777 (PMC7760155; doi:10.3390/foods9121777)
Supplement: Supplementary file 1 [file foods-09-01777-s001.pdf]

S.1. Supplementary data

Supplementary Figure

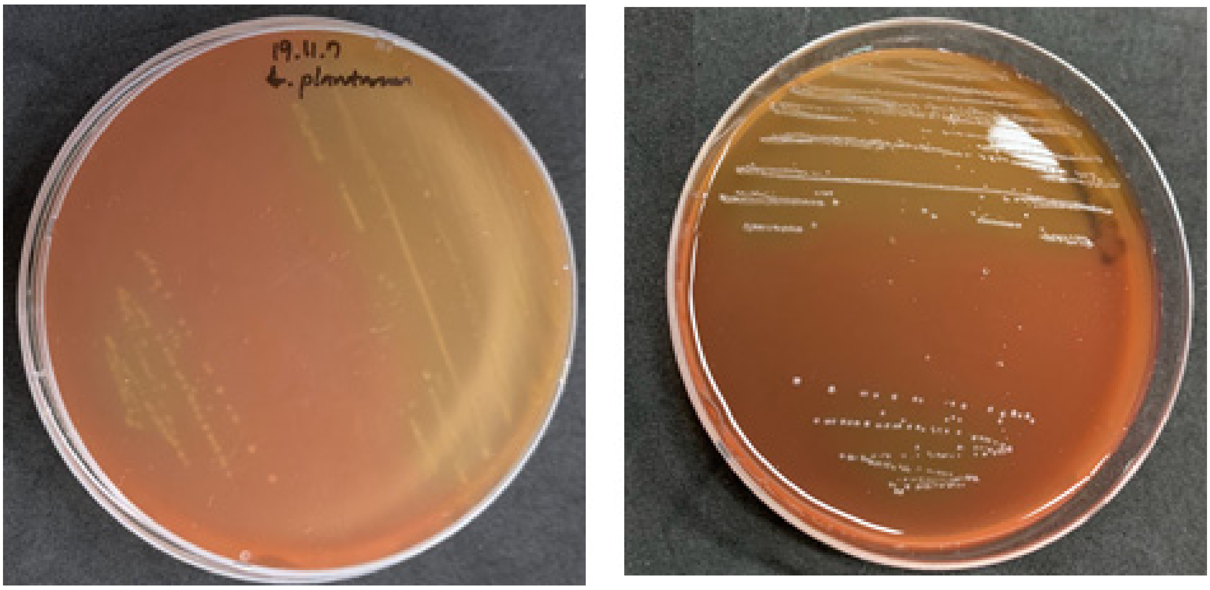

Figure S1. Hemolytic activity of LPLB5.

The hemolytic activity of LPLB5 was investigated by culturing the organism on Colombia sheep blood agar at 37°C for 24 h under aerobic conditions. Clear zones around the colonies (indicating β-hemolysis) were not observed.
